# The Mallet in Filling Teeth

**Published:** 1870-01

**Authors:** 


### Editorial.

### THE MALLET IN FILLING TEETH.

In filling teeth with the mallet, the aim should be to condense the gold to the desired extent, with the least possible jarring of the tooth and its surroundings. Our motto should be, "DRIVE THE GOLD, BUT SPARE THE TOOTH."

How?

The force with which the mallet strikes the plugger is its "momentum." The speed at which it moves in making the stroke is its "velocity." The momentum of each stroke is to be estimated by multiplying the velocity by the weight of the mallet. A mallet weighing one ounce exerts as much force on the plugger as one weighing two ounces, if the velocity of the stroke is doubled.

The force of the stroke is not imparted to all the particles of the plugger at once. It acts first on those struck, and extends from these to the rest. A bullet may be shot through a pane of glass, making but a small round hole in it, while the entire pane may be broken to fragments, by a gentle blow of the same momentum. In the first case, the force of the blow has not time to extend itself, in the brief period required for the bullet to pass through the glass. Consequently, the particles immediately in front of the ball receive its whole momentum, and are torn from their connection. The handle may be driven into a hammer, by brisk blows on its distal end, while blows of the same momentum, with a heavier instrument, will not accomplish the same result. The heavy instrument drives both the hammer and the handle, while the light one, with equal momentum, drives the handle so promptly that it passes in before the motion is diffused to the hammer. The same principle applied to driving the plugger into the gold—that the gold may be driven before the force has time to be diffused to the tooth—requires that the momentum be gained by the combination of a light mallet with strokes of high velocity. With low velocity, much of the momentum extends to the tooth and its surroundings, and all that is so extended is lost on the gold. Practically, then, it requires more momentum to condense the gold with a heavy instrument, than with a light one, and this in addition to the fact that with equal momentum, the tooth is more jarred by the former than by the latter. Then, a light mallet with a long handle, in the hand of an accurate striker, is essential to the best results.

The texture of the mallet is also important.

Let two balls of soft clay or putty, equal in weight, be suspended in contact. Draw one of them aside, and let it fall against the other, and it will drive it forward, and *go with it.* These balls are inelastic. Let the same be tried with elastic balls, and let us not select soft rubber, for that is not highly elastic. Ivory is much more so, hence, let the balls be ivory. The one drives the other forward, as before, but it *does not go with it*, but is instantly arrested in its progress. The more sharply elastic the material of the mallet, the better is the result, then. Or, to sum up, the mallet should be made of highly elastic material, not of soft wood, leather or similar substances, not faced with soft rubber or springs, not made of a soft metal; it should be as light as will answer the purpose; it should have a long handle to insure the needed velocity, should be wielded by an accurate striker; and all this, if it be desirable to *drive the gold*, and spare the tooth. W.

